# Porous spherical gold nanoparticles *via* a laser induced process

**DOI:** 10.1039/d2na00396a

**Published:** 2022-09-02

**Authors:** Gabriele Schmidl, Marc Raugust, Guobin Jia, Andrea Dellith, Jan Dellith, Frank Schmidl, Jonathan Plentz

**Affiliations:** Leibniz Institute of Photonic Technology (Leibniz IPHT) Albert-Einstein-Straße 9 Jena 07745 Germany gabriele.schmidl@leibniz-ipht.de +49 (0) 3641 206299 +49 (0)3641 206438; Friedrich Schiller University, Institute of Solid State Physics Helmholtzweg 5 Jena 07743 Germany

## Abstract

Nanoparticles consisting of a mixture of several metals and also porous nanoparticles due to their special structure exhibit properties that find applications in spectroscopic detection or catalysis. Different approaches of top down or bottom up technologies exist for the fabrication of such particles. We present a novel combined approach for the fabrication of spherical porous gold nanoparticles on low-cost glass substrates under ambient conditions using a UV-laser induced particle preparation process with subsequent wet chemical selective etching. In this preparation route, nanometer-sized branched structures are formed in spherical particles. The laser process, which is applied to a silver/gold bilayer system with different individual layer thicknesses, generates spherical mixed particles in a nanosecond range and influences the properties of the fabricated nanoparticles, such as the size and the mixture and thus the spectral response. The subsequent etching process is performed by selective wet chemical removal of silver from the nanoparticles with diluted nitric acid. The gold to silver ratio was investigated by energy-dispersive X-ray spectroscopy. The porosity depends on laser parameters and film thickness as well as on etching parameters such as time. After etching, the surface area of the remaining Au nanoparticles increases which makes these particles interesting for catalysis and also as carrier particles for substances. Such substances can be positioned at defined locations or be released in appropriate environments. Absorbance spectra are also analyzed to show how the altered fractured shape of the particles changes localized plasmon resonances of the resultingt particles.

## Abstract 2

Nanoparticles consisting of a mixture of several metals and also porous nanoparticles due to their special structure exhibit properties that find application in spectroscopic detection or catalysis.

## Graphical abstract

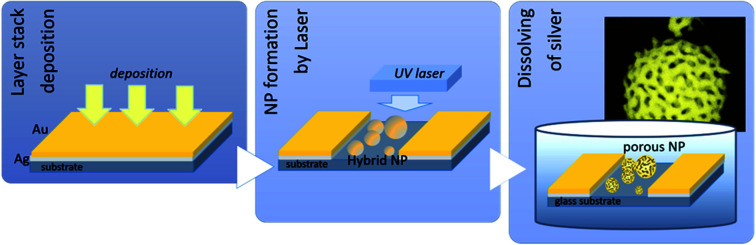

## Introduction

Metallic nanoparticles benefit from their specific optical properties with respect to the generation of localized surface plasmon resonance (LSPR) or in general because of their possible use in applying field enhancement by particles or by nanostructures in the field of surface enhanced or tip enhanced Raman scattering (SERS and TERS). This performance is utilized in chemical or biomedical applications *e.g.* for the detection of bindings or species.^1–5^ The LSPR enhancement effect can also boost several properties of photocatalytic processes. For the plasmonic enhancement of catalytic reactions, alloy nanoparticles with specific metal compositions as well as specific surfaces of the metal nanoparticles play an important role. Increasing photocatalytic efficiency has gained importance in recent years.^6^ A specific surface can be achieved with porous nanoparticles in particular or porous surfaces in general, which show a high surface-to-volume ratio and a low density. These properties make them interesting for many areas of application such as catalysis, optical detection methods or biomedical applications.^7–9^

The starting materials for manufacturing porous metals as particles or surfaces, as is used in the present item, are alloys from which the less noble metal is dissolved out.^7,10,11^ For example, in, ref. 11–13 the authors fabricated gold (Au) and hybrid Au/Al_2_O_3_ porous nanoparticles (NPs) *via* a furnace annealing process at 850 °C or 900 °C and a subsequent dealloying process. In the described context, a selective removal or etching of one component (Ag) by dissolving it out was used as well. The applied dewetting process for particle fabrication in a furnace has the advantage of easy processing, but requires a long time and an atmosphere as inert as possible. Large areas can be processed and mostly a wide size distribution of nanoparticles from several tens of nm to μm can be produced. Furthermore, metals are well miscible at high temperatures. The formation of alloys requires the melting of metals, which limits the use of certain substrates such as low-cost Borofloat glass, that has a lower melting point than a metal. However, this furnace annealing process is only one of a large number of different methods for nanoparticle (NP) fabrication which are divided into top down and bottom up methods.^14^

Among these methods, the chemical synthesis route allows fabricating very small particles, mostly as pure metal particles with or without shells and different shapes such as triangles, cubes, rods or spheres. Such processes require complex synthesis steps and the particles must be stabilized in solution with surfactants that prevent clustering.^15–17^ Thin film structuring by lithography processes, *e.g.* e-beam, photo or sphere lithography, can also be used for particle fabrication. In these cases, the film or the obtained particle composition depends on the initial target material mixture during the layer deposition. The resulting particles are well shaped by the lithography process, are available as bi-metallic particles and do not need a chemical shell to be well separated in contrast to the chemical synthesis, where the particles in the liquid must be prevented from clustering.^18–20^

Another new high-potential synthesis route for pure or multi-component nanoparticle fabrication represents the laser synthesis of colloids^21,22^ or the NP fabrication by Nd-YAG or excimer lasers *via* dewetting or melting of layer systems or ablation.^23,24^ In contrast to colloidal synthesis, this laser induced method allows fabrication of spherical single alloy particles under ambient conditions without time consuming and complex chemical synthesis steps, which adhere on low-cost glass substrates and do not require a chemical shell to avoid agglomeration.

The UV laser process used here deals with the transformation of layers into particles by means of laser irradiation under ambient conditions. Based on the initial layer system, particles in pure form as well as alloy particles can be manufactured by laser treatment. They are generated directly on the substrate and adhere well. The dewetting or melting process in the ns range using one or more laser pulses can be easily up-scaled for large area fabrication of nanoparticles. By changing the laser parameters such as energy density, number of pulses, the pulse repetition rate and the substrate material, individual layer thicknesses, deposition parameters as well as the layer material, the size and the size distribution of the manufactured spherical particles can be well controlled. These extensive relationships have been demonstrated in various literature studies and previous publications for different material systems.^23–28^ A material mixture in particles based on a multi-layer system deposited on a substrate is very well realizable with this method, since excimer laser irradiation is characterized by fast heating, up to melting or evaporation in the ns range. The fast cooling rate can suppress phase separation during solidification. Due to fast processes and the low optical penetration depth, the method also enables the use of Borofloat glass substrates, whose softening point is about 580 °C. The laser just heats up only the above metal layers up to the melting or evaporation (*T*_m_(silver) = 961.8 °C, *T*_m_(gold) = 1064 °C, under normal pressure), while the bulk substrate with its low thermal conductivity remains unaffected.

For LSPR sensing with nanoparticles, as a field of application in biomedical or as a characterization method, the tuning of the resonance position and a narrow spectral width is essential. But a broad absorption enhancement is also of interest in the case of plasmon enhanced photocatalytic applications. Localized surface plasmon resonances can be observed in the scattered light or absorption spectra when nanoparticles are exposed to electromagnetic fields. These resonances, *i.e.* the resonance position and shape, are formed depending on the size, distance of the nanoparticles, the surrounding medium or on the special shape.^6,29–31^ The spectral description in combination with a topological analysis can be used for characterization of the resulting nanoparticles.

In this article, the authors intend to show that gold nanoparticles with porous surfaces can be fabricated successfully based on a fast laser induced particle formation process and a subsequent selective wet chemical etching process, and that the laser parameters have a key influence on the results. This laser processing can be applied to other miscible thin film systems with high melting temperature materials, and it can be widely used for various material systems also on low cost substrates.

## Experimental

### Thin film preparation

All layers were sputter coated. The deposition of the silver (Ag) thin films was exhibited by DC-magnetron-sputtering onto 3 mm thick and one-inch squared Borofloat glass substrates (thermal conductivity *σ* = 1.2 W m^−1^ K^−1^, specific heat capacity *c*_p_ = 0.83 kJ kg^−1^ K^−1^, and transformation temperature *T*_g_ = 525 °C) with a rate of 0.4 nm s^−1^. The argon gas pressure in the deposition chamber was 5 × 10^−3^ mbar. A background pressure of approximately 1 × 10^−5^ mbar was used in the framework of the present experiments and the sputtering power was kept at 25 W.

For the laser induced NP formation tests, different gold (Au) layers with different thicknesses were sputtered whereby the argon gas pressure in the deposition chamber was fixed here at 2 × 10^−2^ mbar and a background pressure of less than 1 × 10^−5^ mbar was used. It worked with a current of 100 mA and a voltage of approximately 270 V resulting in a deposition rate of 1 nm s^−1^. A small number of gold layers used for experiments to generate porous NPs were additionally prepared using a DC sputter coater, and the deposition rate was kept at 0.3 nm s^−1^.^32^

The film thickness of all individual films was fixed between 5 and 20 nm, respectively. The film thickness ratio of Ag and Au was mainly varied between 1 : 1, 1 : 2, and 2 : 1 (Ag : Au). The investigated total film thickness was set at between 15 and 30 nm. The glass substrates were cleaned before deposition using a 3% Deconex bath with ultrasonication and subsequently rinsed with ultrapure water. This procedure was followed by cleaning with acetone and isopropanol in an ultrasonic bath. The samples were then dried with N_2_.

### Laser annealing

The laser setup consists of a pulsed KrF excimer laser, focusing optics, an intensity attenuator, a beam profile homogenizer and an adjustable sample holder for sample positioning. The excimer laser (LPX305, Lambda Physik) emits non-polarized light and operates at a wavelength of 248 nm with a pulse duration of 25 ns. The pulse-to-pulse repetition rate was set to 1 Hz. The laser induced experiments were performed with energy densities of 150 mJ cm^−2^ and 250 mJ cm^−2^, tuned by the intensity attenuator. The number of laser pulses for particle fabrication was 1, 2, 5 and 10. The laser spot was formed to be about 4 × 4 mm^2^ and showed a top hat intensity profile created by the focusing optics and the profile homogenizer. The sample position is achieved using an XY-translational stage.^24,28^

### Selective chemical etching

For the etching process of the mixed Ag/Au particles, a solution of 65% HNO_3_ + H_2_O with a volume ratio of 1 : 2 (*e.g.* 5 ml 65% HNO_3_ + 10 ml H_2_O) was used. The etching or Ag dissolving time was an approximate average of 5 min at room temperature and is one of the parameters for porosity. After etching in a ceramic vessel, the substrate is immediately immersed in ultrapure water (18.2 MΩ cm prepared by a Milli-Q device) and then rinsed several times carefully in ultrapure water to remove the etching solution. The residual Ag and Au layers outside the laser treated areas are completely removed. During this procedure particles remain only in the spot areas.

### Characterization methods

The formed particles were observed by field-emission scanning electron microscopy (FESEM, FEI Helios NanoLab G3 UC, ThermoFisher Scientific). An accelerating voltage of 5 kV was used. The EDX spectra recording was also realized by FESEM with a JSM-6700F from JEOL Ltd using a silicon drift detector (SDD) XFlash 5130 from Bruker Nano. In the JSM-6700F, the energy of the exciting electrons was set to 10 keV and the accumulation live time was 200 s in all cases. To prevent charging effects in the SEM, a 5 nm thick carbon layer is deposited on the sample before analysis.

For optical characterization of the mixed particles after laser treatment as well as after the etching procedure, the transmission spectra were determined using a spectrometer (H2000, Ocean Optics) setup with a coupled fiber (wavelength range of 200–1100 nm) under transmitted illumination with a halogen-lamp (400–900 nm emission and illumination diameter 2 mm) and with a UV-vis spectrometer with an integrating sphere of PerkinElmer in the same spectral range (illumination area 1 × 2 mm^2^). All raw spectra are corrected by the background and light source signal.

The resonance spectra of single pure Au and Ag NPs were calculated depending on different diameters in the wavelength range of 350 to 900 nm using the open source software Mie-Plot (https://www.philiplaven.com/mieplot.htm).

## Results and discussion

### Laser fabrication of Ag–Au nanoparticles

The basis for a two-step manufacturing (laser treatment and wet chemical etching) of porous gold nanoparticles (PG-NPs) demonstrated in this publication is a laser process. For this purpose, the individual pure elements silver and gold are deposited as thin layers on a glass substrate one after the other. Afterwards they were pulse irradiated with a UV laser at 248 nm. During the laser pulse, the film absorbs the optical energy and will be heated up. The laser irradiation can lead above a certain fluence threshold to melting and at still higher fluences, to evaporation. The nanoparticle formation during this short laser process is directly influenced by the laser parameters such as the number of pulses and laser energy density as well as by the material properties such as the type of material, layer thickness (the total thickness or thickness of each layer) and layer order in the case of a multilayer system. The Borofloat glass with an absorption edge at approximately 350 nm has poor thermal conductance and can therefore be an influencing factor for the interaction of the laser and thin film systems and thus for the NP formation, especially if the layer thickness is in the range of the optical penetration depth (see Table 1) because the heat can be piled up in the film.

**Table 1:** Summary of material parameters of Au and Ag under standard conditions, refractive index (*n*), extinction coefficient (*κ*), absorption coefficient (*α*), optical penetration depth (*l*) at *λ* = 248 nm (*τ* = 25 ns) as well as thermal conductivity (*σ*), melting temperature (*T*_m_), thermal diffusivity (*k*), density (*ρ*), specific thermal capacity (*c*_p_) and thermal penetration length *L*_th_.^33–36^

	Gold (Au)	Silver (Ag)
*n*	1.33	1.31
*κ*	1.62	1.39
*α* [cm^−1^]	8.22 × 10^5^	7.03 × 10^5^
*l* = *α*^−1^ [nm]	12	14
σ [W (K^−1^ m^−1^)]	310	410
*T*_m_ [K]	1337	1234.9
*k* [cm^2^ s^−1^]	1.27	1.74
ρ [g cm^−3^]	19.3	10.5
*c*_p_ [J kg^−1^ K^−1^]	129	236
*L*_th_ [μm]	2.5	2.9

**Fig. 1:**
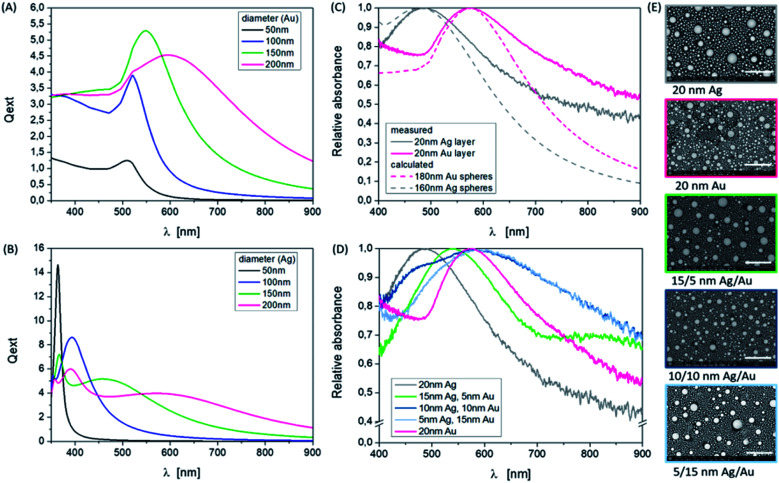
Influence of the Ag and Au composition ratio on the resonance peak position. (A and B) Calculated resonance spectra of single, spherical, and monodisperse Au and Ag NPs with different diameters in the wavelength range of 350 to 900 nm, and the surrounding media is air with *n* = 1 (http://www.philiplaven.com/mieplot.htm). (C) Normalized measured absorbance spectra of pure Au and Ag NP samples based on 20 nm thick single layers using 250 mJ cm^−2^ and 10 pulses in comparison to calculated spectra of single spheres in the wavelength range of 400 to 900 nm. (D) Variation of the thickness ratio of Ag/Au in comparison to the pure materials. (E) SEM images (BSE) of the fabricated nanoparticles corresponding to (C). Scale bars are 1 μm, magnification ×50 000, and HV = 5 kV.

Especially here, we want to show the influence of the material thickness ratio for the bi-layer system Ag/Au and also of a selected energy density and pulse number of the laser on the observed particle sizes and distributions. As an example, Fig. 1 demonstrates the influence of the individual layer thickness for a fixed overall thickness on the particle distribution and on the plasmonic response for a specific, favorable energy density of 250 mJ cm^−2^ and a defined layer sequence (Ag: bottom layer; Au: top layer) using 10 laser pulses. The results represent different shapes of absorbance curves with different peak positions. The laser irradiation of pure silver and gold layers with the same thickness results in similar curve shapes, which means similar NP sizes and size distributions. The energetic position of the plasmon resonance is influenced not only by the material type (number of free electrons) but also by the NP size, shape and the dielectric environment of the particles, which is air (*n* = 1) and the substrate (Borofloat, *n* = 1.47) in the investigated experimental case. The position of the measured absorbance peak for silver NP was exhibited at 480 nm and was identified at 580 nm for gold NPs. This corresponds to an average size of 160 nm or 180 nm, respectively (Fig. 1C), determined by simulations. The performed simulations of the scattering efficiency *vs.* the wavelength using the open source software MiePlot, which is based on the BHMIE algorithm for Mie scattering from a sphere,^37^ are valid for individual spherical NPs under an air environment (*n* = 1). The obtained extinction efficiency curves for Ag and Au are shown in Fig. 1A and B. The dielectric functions of Au and Ag in particular can be found for example in ref. 36,38,39 Furthermore, the influence of the substrate was not to be considered in the performed simple calculations based on the software. But this fact plays an important role in the exact determination of resonance positions and shapes, just like the spacing of the particles. A possible approach to include the substrate effect is to use an effective refractive index which considers all surrounding media. This allows a more accurate comparison of simulation and measurement and is described in ref. 40. The investigation of these influences will be the subject of further work, since other highly refractive substrate materials such as SrTiO_3_ (*n* = 2.4) are also of interest. The broadening of the measurement curves in contrast to the simulation can also demonstrate the inhomogeneous size distribution by the formation of higher order modes in the spectra, since the spectra comprise a superposition of a large particle number with quite different sizes. Additionally, in the SEM images (Fig. 1E) bi- or multi-modal size distributions can be identified, which are a bit clearer to see for pure silver particles. In the case of pure silver, the maximum particle diameter (*d*) is about 400 nm, the smallest particles have a size of *d* = 10–20 nm and as seen in the SEM image a large number of particles show a diameter of 80–90 nm. The gold particles have a slightly different size distribution on the investigated surface area. A few large particles with a diameter of about 300 nm can be observed, and also many small particles with diameters ranging from 10 nm to 100 nm can be observed. To determine the particle sizes, the image processing software ImageJ (https://imagej.nih.gov/ij/) can be used, for example. The spectra of the mixed systems are a bit more complex because they show a superposition of resonances of both materials and particle sizes (Fig. 1D). The less silver is contained in the system, the more dominant the resonance of gold will be.

**Fig. 2:**
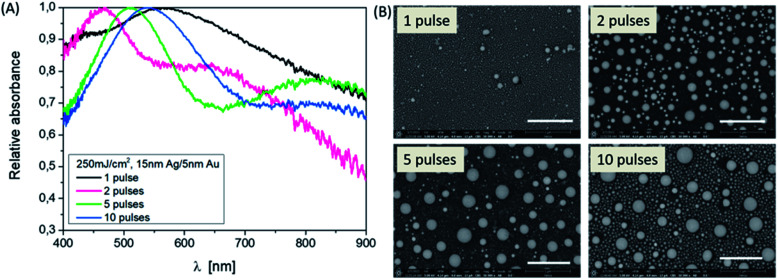
Influence of the pulse number on the particle formation, Ag–Au ratio: 3 : 1. (A) Absorbance spectra of the samples in the wavelength range of 400 to 900 nm. (B) SEM images (BSE) of the produced nanoparticles using 250 mJ cm^−2^, 10 pulses corresponding to (A). Scale bars are 1 μm, magnification ×50 000, and HV = 5 kV.

**Fig. 3:**
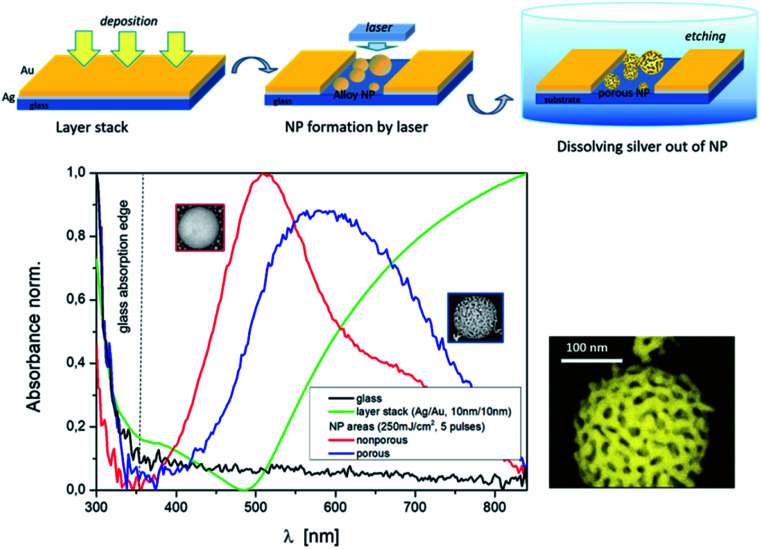
Top: sequence of the process to fabricate porous NPs and bottom: absorbance/resonance spectra of layer stack Ag/Au (10/10 nm–green curve) in comparison to laser treated areas (250 mJ cm^−2^, 5 pulses) with nonporous NPs (red curve) and with porous NPs (blue curve), and a magnified illustration of a porous NP.

The influence of varying the number of pulses at a fixed energy density on the resulting NPs can also be identified at a defined layer thickness of 15 nm Ag/5 nm Au. In Fig. 2, this is clear from the shape of the absorbance curves, but by specifically looking at the position of the band's maximum, corresponding to the SEM images considering the sample 15/5 nm Ag/Au layer. Applying one laser pulse, resonance peaks at about 420 nm and 550 nm due to Ag and Au NP structures, are forming and can be observed. Since the absorption edge of gold is at about 510 nm, resonance peaks smaller than this wavelength can be assigned to silver (absorption edge at about 380 nm, see also Fig. 3). The modulation of the curve changes as the number of pulses increases. The short wavelength resonance moves to the longer wavelength side and becomes broader and a higher mode emerges which is less pronounced in terms of intensity.

These correlations with respect to the number of pulses demonstrate that it is possible to control and influence the generation of nanoparticles by means of laser radiation with large numbers of parameters. The basic mechanism of NP forming is the interaction of the laser and thin films, whose thickness is here in the range of the optical penetration depth or absorption length (*l*(*λ*) = 1/*α*(*λ*) = *λ*/(4π·*κ*(*λ*)), *α*(λ)) – absorption coefficient, *κ*(*λ*) – extinction coefficient of the individual materials at the wavelength *λ* = 248 nm. If the layer thickness increases, the thermal conductivity (*σ*) of the metals or the thermal diffusion length (*L*_th_ = (*2kτ*)^1/2^) with *k* = *σ*/(*ρ*·*c*_p_) as thermal diffusivity and *τ* – pulse length enters the process and thus influences the threshold for melting at higher energies for evaporation. These parameters are summarized also in Table 1.

In ref. 27 a linear dependence of melting and evaporation thresholds on thickness was described as long as the thickness is larger than the optical absorption depth and smaller than the thermal diffusion length of the film (α^−1^ < *d* < *L*_th_). For metals, *L*_th_ ≫ *α*^−1^ is generally valid. If the layers are very thin, it should also be noted that due to island growth, some of the layers are not closed already after deposition. That can explain different behaviors upon laser exposure than when using thicker layers.

### Porous Au nanoparticles

The porosity of the Au nanoparticles was generated *via* a selective chemical dissolved out of Ag after the above described laser treatment. The nanoparticles exhibit open pores after using the wet chemical etching process (Fig. 3, top). The formation of such pore structures in NPs has been demonstrated for instance in ref. 12. However, upstream of the particle production process there was a furnace process. In ref. 41 a model of porosity evolution for the fabrication of three-dimensional porous material networks by dealloying was presented, including different approaches based on thin films. Basic processes are dissolution and diffusion of atoms.

**Fig. 4:**
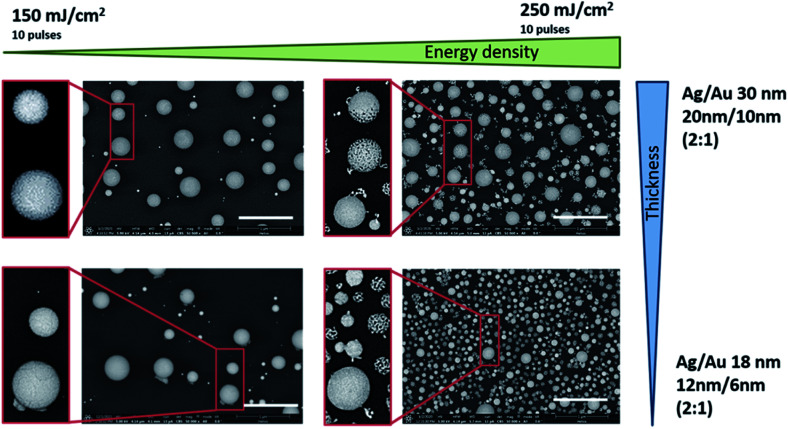
SEM images (BSE) of nanoparticles, after laser irradiation and dissolving silver. Layer stack ratio: 2 : 1, total layer thickness: 30 nm and 18 nm, different laser energies: 150 and 250 mJ cm^−2^, and 10 laser pulses. Scale bars: 1 μm, magnification: ×50 000, and HV = 5 kV.

Fig. 3 (bottom) demonstrates the change in the absorbance spectrum of a thin bi-layer film (green curve) after nanoparticle laser fabrication (red curve) and the influence on the resonance curve after the formation of porous nanoparticles (blue curve). Below 350 nm the absorption edge of the Borofloat glass (black curve) is evident. Etching parameters that can influence the porosity are the composition of the etching solution and the etching time. But to show the principle combination of a special laser process and a subsequent etching or dissolving or removal of silver out of the mixed Ag/Au nanoparticles, in the first step, we fixed the etching parameters and focused on the variation of the laser parameters and film thicknesses. This parameter variation allows the following statements, as shown in Fig. 4.

Larger NPs and a higher number of these NPs result from the laser process at a higher total layer thickness (30 nm in total) in the ratio 2 : 1 Ag/Au. For the same thickness ratio but a smaller total layer thickness (18 nm in total), the particles from the laser process are smaller and arise in absolutely larger numbers, and both phenomena can be observed especially when using the higher energy density of 250 mJ cm^−2^ (Fig. 4, top right and bottom right). For an etching time of 5 min, the individual very small NPs disintegrate into thread-like structures or partially dissolve completely as a thread (see image detail Fig. 4, 250 mJ cm^−2^ and Ag/Au layer of 18 nm thickness). The large particles in the range of approximately 200–300 nm, on the other hand, show either a completely porous network, are partially etched, or exhibit only surface etching (see image detail Fig. 4, 250 mJ cm^−2^, and Ag/Au layer of 30 nm thickness). The cause may be a too short etching time but it’s more likely a higher inhomogeneously distributed silver content. However, the etching results also show that although the silver in the entire particle is mixed during the melt, the solid solution formation and thus the mass fractions in the different sized particles and at different positions within a single particle during the cooling process differ from each other also depending on the initial mass ratio. It can be assumed that very small particles solidify faster than large ones. But all particles react to the silver removal and changed their surface structure more or less as a 3-dimensional network. In general, however, it can be said that the resulting network uses a specific reaction process during laser melting and that the dissolving process shows its particularity here compared to a furnace process.

**Fig. 5:**
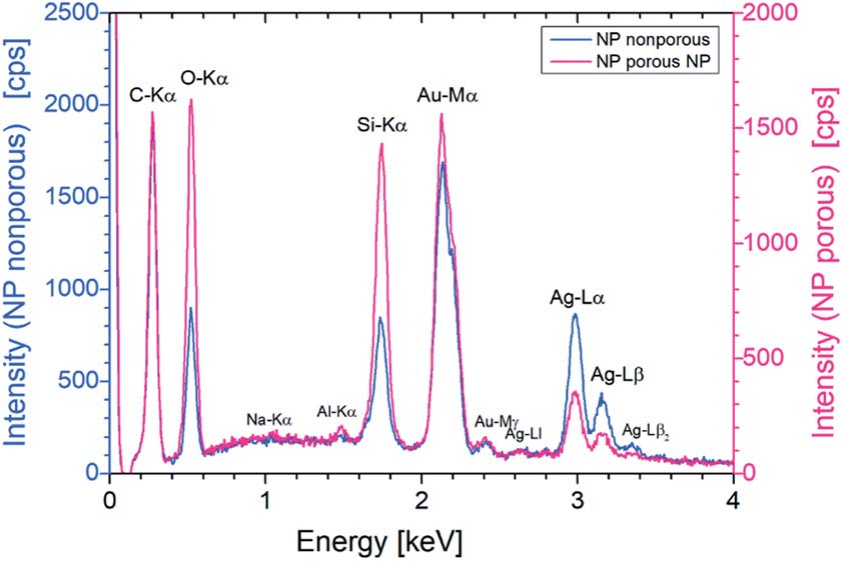
EDX spectra of a single porous and nonporous NP. The energy of the exciting electrons was set to 10 keV and the accumulation live time was 200 s. Both particles have approximately the same size (approx. 300 nm).

Energy dispersive X-ray (EDX) analysis was used to show the reduction of silver in individual particles. It has been confirmed that the etching process reduces the silver content (Fig. 5). The study of the intensities of the Ag Lα line and the Au Mα line resulted in a ratio of 1 : 2 for a non-porous particle. For a porous NP, however, the ratio is 1 : 5. Thus, for a nonporous particle a proportion of gold and silver analogous to the material ratio of the initial layers was found and after etching for 5 min gold with approx. 81% by mass and Ag with 19% by mass can be assumed. In the case of smaller particles, which almost completely dissolve or become highly porous during the process, the silver content is further reduced.

**Fig. 6:**
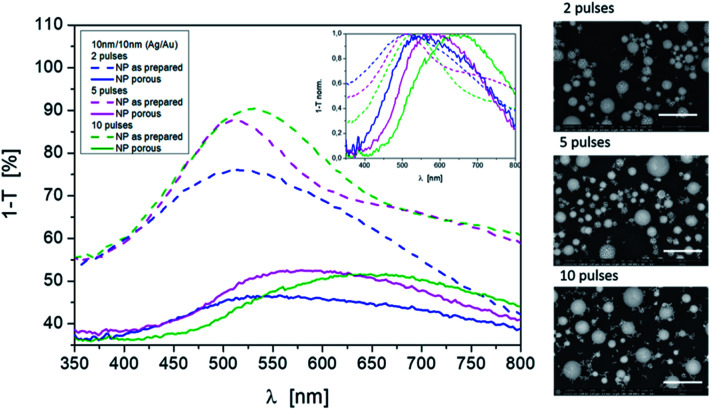
Influence of pulse numbers on the resonance behavior for mixed nonporous as prepared NPs and porous NPs after dissolving silver. Laser energy density: 250 mJ cm^−2^; pulses: 2, 5, and 10; initial material ratio: 10 nm Ag/10 nm Au. SEM images (BSE): Scale bars are 500 nm, magnification ×100 000, and HV = 5 kV.

In addition to the laser energy and the layer thickness, different pulse numbers (one pulse after another) are also responsible for the formation of mixed nanoparticles with different size distributions and compound states. The difference is particularly evident in the comparison of one or two laser pulses and ten laser pulses per area. Consequently, different porosities result even after etching for a fixed process. This is also shown in the resonance spectrum (Fig. 6). When looking at the position of the resonances on their own, a redshift with the increasing pulse number can be observed after the Ag dissolving out process. The half-widths of the curves are similar. The mode behavior blurs.

## Conclusions

We have demonstrated a new and fast approach for the fabrication of porous Au nanoparticles on low cost glass substrates, which uses a laser induced nanoparticle preparation process. The laser process benefits from very fast heating, up to melting or evaporation in the ns range, with an excimer laser, and from fast solidification contrary to an oven dewetting process. The laser parameters such as the energy density and pulse number as well as the layer thickness of the individual Ag and Au layers in the bi-layer system can be used to adjust the sizes and size distributions of the spherical nanoparticles in the particle formation process before removing the silver.

The subsequent wet chemical etching process with diluted nitric acid results in a 3-dimensional nano-network. At the same time, fine network structures can be found in the complete particle or in parts of a particle, and also worm-like structures can be found in small particles. It can be assumed that this is due to the special compounding process during laser melting because the results are dependent on the laser parameters and thus on the particle size, size distribution and mixture. Here the process of silver dissolving out for porous nanoparticle formation shows its particularity compared to a long-time furnace process.

The formation of localized surface plasmon resonances by the hybrid particles depending on the laser energy and pulse number was investigated. Their change in resonance position and shape after the etching process was presented. However, both processes, the laser process and the etching process, must be further investigated, especially to better understand the influence of melting and solidification on the dissolving out process. This novel approach to maintain a fine-structured porous nano-network in spherical Au nanoparticles is very interesting for the use of such particles in catalysis and also as carrier particles for substances.

## Conflicts of interest

The authors declare no competing interests.

## Supplementary Material
